# The Role of Nature and Nurture for Individual Differences in Primary Emotional Systems: Evidence from a Twin Study

**DOI:** 10.1371/journal.pone.0151405

**Published:** 2016-03-21

**Authors:** Christian Montag, Elisabeth Hahn, Martin Reuter, Frank M. Spinath, Ken Davis, Jaak Panksepp

**Affiliations:** 1 Institute of Psychology and Education, Ulm University, Ulm, Germany; 2 Department of Psychology, University of Saarbrücken, Saarbrücken, Germany; 3 Department of Psychology, University of Bonn, Bonn, Germany; 4 Center for Economics and Neuroscience, University of Bonn, Bonn, Germany; 5 Department of Psychology, The University of North Carolina at Charlotte, Charlotte, North Carolina 28223, United States of America; 6 College of Veterinary Medicine, Washington State University, Washington 99164, United States of America; Shinshu University School of Medicine, JAPAN

## Abstract

The present study investigated for the first time the relative importance of genetics and environment on individual differences in primary emotionality as measured with the Affective Neuroscience Personality Scales (ANPS) by means of a twin-sibling study design. In N = 795 participants (n = 303 monozygotic twins, n = 172 dizygotic twins and n = 267 non-twin full siblings), moderate to strong influences of genetics on individual differences in these emotional systems are observed. Lowest heritability estimates are presented for the SEEKING system (33%) and highest for the PLAY system (69%). Further, multivariate genetic modeling was applied to the data showing that associations among the six ANPS scales were influences by both, a genetic as well as an environmental overlap between them. In sum, the study underlines the usefulness of the ANPS for biologically oriented personality psychology research.

## Introduction

The study of primary emotional systems represents an important research endeavor to better understand psychological well-being and psychopathologies such as affective disorders in humans [1]. Specifically, it has been put forward that imbalances in these ancient emotional brain systems go along with psychopathologies, e. g. that a lack of PLAY behavior in childhood might be linked to Attention Deficit Hyperactivity Disorder (ADHD) later on or that an overactivation of the SADNESS (separation-distress, psychological-pain) system and the subsequence reduction of SEEKING urges are major cause for depression (for full discussion, see [2, 3]). (Primary emotional systems are printed in capital letters, as a formal designation for primal emotional systems of mammalian brains, partly intended to distinguish them from the vernacular emotional terms commonly used in emotional and other psychological research. The need for scientifically clear designators for primary-process (i.e., evolved) brain emotional and motivational systems is essential, and the formal designators should help avoid mereological fallacies (part-whole confusions) which are abundant in neuropsychological discourse (see [4]). A major goal of Panksepp’s *Affective Neuroscience* perspective has been dedicated to elucidating how primal (i.e., evolved) neuropsychobiological emotional networks underlie core affective processes (using animal models to illuminate foundational human affects), and how their upward influence in the brain shape diverse higher-order psychological and behavioral processes. By applying techniques such as deep (subcortical) electrical stimulation of the mammalian brain and pharmacological challenges his group has provided evidence for seven distinct primary emotional systems (SEEKING, RAGE, FEAR, LUST, CARE, PANIC and PLAY) anchored in phylogenetically old brain areas which not only instigate instinctual emotional behaviors, but also influence and control the secondary processes of learning and memory and tertiary-process such as cognitive decision making [1]. These primal emotions are survival systems, which with various sensory and homeostatic (e.g., HUNGER and THIRST) affects constitute the primal value (reward and punishment) systems of the brain. These subcortical systems are foundational for higher mental processes in all animals since extensive damage to such systems compromise consciousness, and they are envisioned to guide the development of higher mental processes, including personality dimensions which, with maturation, gradually provide higher reciprocal-regulatory cortical control over lower affective processes.

The mammalian (especially human) prefrontal cortex and other neocortical regions can control emotional outbursts from subcortical areas (providing top-down behavioral and psychological regulation). But in extreme situations—such as in high danger—our brains often respond with stereotypic genetically-anchored affective response patterns (instigating bottom–up arousal of higher-order brain processes) such as strategies for fight, flight or freezing (e. g. [5]), which helped our ancestors to not only escape various hazardous situations but to develop cognitive skills to avoid them in the future (see also a new questionnaire measuring these distinct fear tendencies [6]). So different primary/basic emotions have different functions with respect to survival and reproductive behaviors. In the end a better understanding of the functioning and interplay of these emotional systems should facilitate development of new therapeutics to better treat a wide range of psychiatric disorders [2,3].

The seven primary emotional systems of Panksepp’s primary-process affective neuroscience can be divided into two larger groups of positive and negative emotions. The emotional systems belonging to the first group of positive emotions are called SEEKING, LUST, CARE and PLAY (in presumed evolutionary order), whereas the latter group representing negative emotions comprises RAGE (also labeled ANGER in discussions of human personality), FEAR (or “anxiety” in the vernacular), and PANIC (namely primary-process separation distress, or higher-order SADNESS, which we deemed a more clear and appropriate designator for human personality profiling). The SEEKING system energizes human beings and helps them not only to be energized with “enthusiasm” and “interest”, in explorative/investigative way in everyday life. The PLAY system has been best characterized not only by the instinctual nature of rough and tumble play in most mammals–a very bodily evolved form of play–best observed in all young mammals, including human childhood, with the brain mapping providing clarification of brain regions where Deep Brain Stimulation (DBS) evokes laughter-type play vocalizations in animal models [7]. The function of the PLAY system probably relates to learning about social structures/hierarchies (e.g., eventual social dominance), learning to cope with losing or being defeated, shaping social-appetitive motoric skills and from a psychological perspective, simply having fun (which may promote bodily and mental health). The LUST and CARE system are of high importance for reproductive success and social bonding and are deeply entwined. The PLAY system is probably evolutionary the youngest with LUST reproduction circuits evolving earlier than the genetic programs for CARE—nurturing other individuals especially one’s own offspring. The FEAR system has been already mentioned above and helps mammals to free themselves from danger. The RAGE/ANGER system facilitates acquiring and holding-on to resources, and can be activated by frustrations (that can arise from higher-order encoding of desires). Finally, the PANIC/SADNESS system reflects arousal of what has traditionally been called “separation distress” the chronic overactivity of which is associated with depression [2,3,8]. For cross-mammalian brain research purposes, this system has been formally designated the PANIC system, which is illustrated by typical panic behaviors and feeling (i.e., separation distress calls, commonly called “crying”) when children get lost and are out of sight of their parents or other caregivers.

Besides the importance of neuroscientific techniques, especially DBS, to study primary emotional systems, Davis et al. [9], published a self-report inventory called Affective Neuroscience Personality Scales (ANPS), updated and refined in Davis & Panksepp [10], aimed at measuring individual differences in these primary emotional systems. The publication of these scales represents an important addition to the toolbox of biologically/behaviorally oriented personality psychologists, because Panksepp’s primary emotional systems could be viewed as being among the evolutionary oldest contributors to human personality (influencing human personality bottom-up development as reflected by their neuroanatomical foundations in the “old-mammalian” and “reptilian” areas of Paul MacLean’s Triune Brain Concept; see also [11]). The ANPS contrasts to classic questionnaires reflecting the Five Factor Model of Personality (e. g. [12]) and may be more appropriate for guiding in the investigation of the biological underpinnings of individual differences in primary sources of temperament, namely one’s genetically controlled emotional strengths and weaknesses. For instance, Montag & Reuter [13] highlight the potential importance of these scales in the context of disentangling the molecular genetics of primary emotional systems and personality. As the Five Factor Model of Personality is based on a lexical (adjective-based) approach it does not help in hypothesizing about diverse neurobiological affect-engendering brain systems that are critical brain substrates underlying human personality. The usefulness of the ANPS for biologically-oriented personality psychology can be best explained by a small example. If animal models show that PLAY behavior in rodents is modulated by opioids (as it is, see [14]), the dynamics of brain opioid systems should also be of relevance for human ludic activities, because these ancient brain systems are highly conserved across species.

As postulated by Turkheimer [15] and newly confirmed within a meta-analysis [16], all human traits are heritable. For the Big Five personality traits, several studies in the past 50 years of research revealed a strong genetic basis for all five personality factors in the range of about 40–60% (e.g. [17]). In terms of environmental contributions, comparable amounts of personality variation can be explained by non-shared environmental experiences. This has also been underlined in a recent meta-analysis [18]. For the ANPS scales, Davis et al. [9] investigated the extent to which self-reports derived from the ANPS questionnaire were related to self-report measures of the Big Five personality traits, i.e. how closely core emotional systems were associated with basic personality traits. Each of the six ANPS scales was found to be closely related to at least one of the Big Five personality scales. The authors concluded that the six core emotional systems assessed by the ANPS scales constituted the roots of adult personality structures, and developmentally contributed to the construction of higher-order emotional traits. Given these findings and the theoretical concept behind the ANPS, one would postulate a strong genetic basis of all the basic emotional systems. With respect to associations among the ANPS scales, which can be depicted by a higher-order positive and negative system, one would further expect a common genetic basis underlying these emotional systems. Please note that LUST was intentionally dropped from the ANPS, because it overlaps greatly with homeostatic affects (e.g., peripheral hormonally-controlled core affects) and because of social reticence or lack of frankness in responding to questions concerning one’s sexuality. Also, such affective responses to one set of questions could potentially create spill-over problems for people responding to other trait questions frankly, but as discussed later, a Spirituality scale was added to evaluate therapeutically-important existential dimensions of existence.

To the best of our knowledge—there are currently no scientific-empirical studies showing the relative contribution of genetic influences on individual differences in these primal emotional foundations of human personality. Hence, the genetic and environmental etiology of individual differences in these traits as well as the etiology of associations among these systems remains poorly understood. Given this fact, the present study aimed to quantify for the first time, the relative influence of both nature and nurture on individual differences in primary emotional systems by means of identical (monozygotic) and fraternal (dizygotic) twin study. Univariate and multivariate genetic modeling was applied to investigate the extent of genetic sources on each emotional system and covariations among them to explore the structural nature of primary emotionality.

## Methods

### Participants

The sample was drawn from the Twin Study on Internet- and Online-Game Behavior (TwinGame), a study of adult twins and non-twin sibling pairs reared together. To realize the twin sample, we reverted to contact information from twins who had participated in previous voluntary German twin studies (e.g. SOEP twin study, ChronoS; for details see [19]. In addition, we invited twins and non-twin sibling pairs (with a maximum age difference of three years) via public announcements to participate in the study. Twins with previous contact information were contacted via telephone and invited to complete an online or paper-pencil version of our questionnaire addressing different areas, such as Internet consumption behavior, personality, health, subjective well-being, empathy and several attitudes. The resulting data set for the present study contained a total of 795 individuals (56% overall participation rate) including n = 303 monozygotic twins (149 complete pairs), n = 172 dizygotic twins (85 complete pairs), n = 267 non-twin siblings (122 complete pairs) and 53 individuals with unknown zygosity. Information on age and the gender distribution is presented in Table 1.

**Table 1:** Demographic characteristics of the total sample and the subsamples of twins and siblings.

Sample	N	N _pairs_	*M*_*age*_	*SD*_*age*_	% Women	% Middle class
Total	795	356	30.2	9.6	72.8	59.7
MZ twins	303	149	33.6	9.9	77.2	63.9
DZ twins	172	85	32.9	10.1	65.3	58.8
Siblings	267	122	23.8	3.9	74.6	52.0

*Note*. *M*_*age*_ = Mean; *SD*_*age*_ = Standard deviation; N = 53 individuals with unknown zygosity.

All participants filled in the Affective Neuroscience Personality Scales (ANPS), as described in the next section. Zygosity was determined through self-reports assessing physical similarity (e.g., eye color, hair structure, skin color) as well as the frequency of twin confusion by different relatives, teachers, and peers across the life span (accuracies in the magnitude of 95%; for details, see [20,21]). The study was approved by the research ethics’ committee of the University of Bonn, Germany.

### Questionnaire

We administered the German version of the ANPS (Reuter, Panksepp, Davis & Montag, test manual to be published at Hogrefe Publishers, soon) containing 110 items ranging from strongly agree to strongly disagree (four-point Likert scale) and reflecting a German translation of the ANPS as published by Davis et al. [9]. The ANPS measures individual differences in all mentioned primary emotional systems with the exception of LUST for the reasons mentioned above. To reiterate, the questionnaire contains one additional scale called Spirituality, which reflects no known primary emotional system, but is included due to its potential psychotherapeutic relevance, (e. g. in the treatment of alcohol addiction). In the present sample, internal consistencies of the German version of the ANPS were satisfying and ranged from .69 (SEEK) to .87 (FEAR) which was in line with the psychometric characteristics reported by Davis et a. [9]. All these parameters are summarized in Table 2. Bivariate phenotypic correlations among the six ANPS scales are presented in Table 3.

**Table 2:** Affective Neuroscience Personality Scales: Means, standard deviations and reliability estimations for the sample and subsamples of twins and siblings.

	*M (SD)*	*M*_*MZ*_ *(SD)*	*M*_*DZ*_ *(SD)*	*M*_*SIB*_ *(SD)*	Reliability
SEEK	38.6 (4.5)	38.5 (4.4)	38.1 (4.2)	39.2 (4.7)	.69
FEAR	35.8 (6.6)	35.3 (6.5)	35.3 (6.6)	36.7 (6.7)	.87
CARE	40.8 (5.2)	40.8 (5.2)	40.2 (5.2)	41.2 (5.4)	.74
ANGER	35.3 (6.0)	35.1 (6.3)	34.5 (5.2)	36.1 (6.1)	.83
PLAY	40.4 (5.3)	40.4 (5.0)	39.6 (5.5)	41.2 (5.5)	.78
SADNESS	33.9 (4.9)	33.7 (4.9)	33.4 (4.5)	34.4 (5.3)	.71
Spirituality	25.8 (5.7)	25.7 (5.5)	25.9 (6.0)	25.8 (5.7)	.81

*Note*. *M* = Mean total sample; *SD* = Standard deviation; *M*_*MZ*_ = Mean MZ twins; *M*_*DZ*_ = Mean DZ twins; *M*_*SIB*_ = Mean siblings

**Table 3:** Phenotypic correlations among ANPS scales (correlations for twin1above the diagonal and correlations for twin2 below the diagonal).

	SEEK	FEAR	CARE	ANGER	PLAY	SADNESS
SEEK		-.22**	.16**	-.14	.32**	-.13
FEAR	-.25**		.07**	.35**	-.38**	.66**
CARE	.22**	.07		-.00	.25**	.21**
ANGER	-.03*	.31**	-.07		-.12*	.32**
PLAY	.40**	-.36**	.37**	.01		-.33**
SADNESS	-.09	.62**	.21**	.26**	-.25**	

*Note*. ANPS scales were corrected for age and sex effects

* = p < .05

** = p < .01.

### Statistical Analyses

First, ANPS scale scores were computed by taking the sum of the corresponding items (in part reverse coded) for each ANPS factor as described by Davis et al. [9]. Prior to behavior genetic modelling, age and sex effects as well as prerequisites for structural equation modelling were inspected for each scale. The perfect correlation for age and sex in same-sex twins can inflate twin similarities [22,23]. To address this potential confounding, raw scores of the ANPS scales were corrected for linear and quadratic sex and age effects as well as interaction effects between sex and age prior to behavior genetic analyses by using multiple regression analyses. Following standard practice, genetic analyses were based on residual scores. Further, we basically used the standard model for twins reared together to decompose the phenotypic variation into its genetic and environmental variance components. The standard twin design is based on several assumptions: First, the equal environment assumption (EEA) assumes that MZ twins share environmental influences to the same degree as DZ twins (e.g., Borkenau, Riemann, Angleitner, & Spinath, 2002). Second, no assortative mating is assumed. Third, there is no gene-environment correlation or interaction (Purcell, 2002). In general, different sources of variance can be considered to explain why individuals differ with respect to certain characteristics and behaviors. On the one hand, individuals can differ because of genetic differences between them or vice versa family members (e.g., twins, siblings) can be similar to each other because they share a certain amount of genetic similarity. The genetic variance indicated as overall heritability can be subdivided into additive genetic influences (commonly denoted as *A*) and non-additive genetic influences, modeled as genetic dominance (commonly denoted as *D*). On the other hand, resemblance between family members can be due to shared environmental experiences contributing to similarity while differences between family members can be explained by different environmental experiences that are specific to each individual and contribute to dissimilarity. Hence, the environmental variance comprises shared (commonly denoted as *C*) and non-shared environmental influences (commonly denoted as *E*). Non-shared environmental influences are usually modeled as residual variance that includes measurement error [24].

In the basic twin model, analyses are based on the comparison of the MZ and DZ twin similarities that is being traced back to the difference in the proportion of segregating genes shared between MZ twins and DZ twins. More specifically, different patterns of MZ and DZ resemblance suggest which influences should be expected to be important. For instance, higher MZ twin correlations than DZ twin correlations are indicative of genetic influences in general because of the higher genetic similarity of MZ twins. MZ twins share 100% of their additive genetic background, while DZ twins (and non-twin siblings) share on average only 50% of additive genetic influences. If the MZ twin correlation is more than twice the DZ twin correlation, there is also evidence of genetic effects due to dominance over additive genetic influences because MZ twins share 100% D influences, while for DZ twins, the dominance component should be about .25. Less than perfect MZ twin correlations (rMZ< 1) suggest non-shared environmental influences, not only developmental-learning but also post-natal epigenetic ones, contributing to this dissimilarity. Comparable high correlations for both MZ and DZ are indicative of shared environmental influences. In the twins reared together model, however, genetic dominance and shared environmental influences are confounded and cannot be estimated simultaneously [25]. Whether shared environment or genetic dominance can be expected in a particular model depends on the pattern of MZ and DZ twin similarities. In the present design, we included a third group of non-twin siblings. Just as DZ twins, non-twin siblings share on average half of their segregating genes (A) and 25% D influences. However, twin and non-twin siblings may differ concerning the impact of shared environment. DZ twins share the same prenatal environment, belong to the same cohort of children and because they are twins there could be something like a “specific twin environment”. Therefore, sources of variation unique to twins might be valid if DZ twins remain more alike than non-twin siblings after genetic effects are accounted for. To investigate twin specific environmental influences, we first specified different shared and non-shared environmental influences for twins (MZ and DZ twins) and non-twins siblings. After fitting this model, we equated twin and non-twin environmental influences and compared the fit statistics to determine the importance of twin-specific environmental influences.

MZ and DZ as well as non-twin sibling variance–covariance matrices were calculated as intra-class correlations (ICCs) and analyzed by fitting genetically informative structural equation models via maximum likelihood using OpenMx [26]. To test for the assumptions of mean and variance homogeneity in the CTD, first, a fully saturated model was tested against a saturated model where means and variances were equated within twin and sibling pairs and across the groups (i.e., MZ, DZ, siblings) for each of the ANPS scales. The same procedure was performed prior to multivariate modeling. We then fitted univariate genetic models for each ANPS scale separately including the test whether twins differ significantly from non-twin siblings as described above. To gain a first insight into possible underlying sources of covariance among the six ANPS scales, multivariate cholesky decompositions [27, 28] were fit to the data. This approach can be used (a) to determine the importance of genetic and environmental influences on associations between variables independent of their influence on other variables and (b) to analyze the extent to which genetic as well as environmental influences on the variables overlap. Further, more restricted and more theoretically driven models, such as different independent and common pathway models, were fit to the data to test for a possible distinction between for example a positive and negative component of basic emotional systems. Within an independent pathway model [29], common genetic and environmental factors can be specified representing shared variance between all ANPS scales or alternately representing a positive and a negative component of emotionality. These common genetic and environmental factors influence the observed variables directly, without an intermediate higher order factor. In addition, scale specific factors are specified. Since the evidence for the existence of a clear distinction between a negative and positive emotional system is scare, additional common pathway models [30] were investigated. The first common pathway model assumes that the phenotypic covariance between all six scales can be explained by a single ‘phenotypic’ latent variable that can be decomposed into genetic and environmental factors. The second common pathway model specifies two phenotypic latent variables, one for SEEK, CARE and PLAY as positive component and one for FEAR, ANGER and SADNESS as negative component. Also, combined independent pathway and cholesky specifications were applied to the data (for similar implementations of these models, see [31]). Given that the cholesky decomposition model is fully parameterized, it can be used as a reference model to evaluate the fits of the more restricted models.

Overall model fit was evaluated by using the *χ*^*2*^-test as well as the Akaike’s information criterion (AIC; [32]). The lower the AIC, the better the fit of the model to the observed data. Due to the limited sample size and hence power considerations, we focus on the results for the full models (ADE and ACE models), instead of reduced models (e.g. AE model without shared environmental influences), given that the exclusion of any genetic or environmental effect may result in biased estimates of the remaining factors in the model, even if the removed factor was not significant [25]. With respect to multivariate model fitting, nested submodels were compared by hierarchic *χ*^*2*^-test. The *χ*^*2*^-statistic is computed by subtracting -2LL (log-likelihood) for the full model from that for a reduced model. We performed model fit comparisons for each multivariate submodel with respect to the full cholesky model as well as the respective full model within the specific type of multivariate model (e.g. within the group of independent pathway models). Given the complexity of the multivariate models, we also observed reduced models (e.g. dropping common or specific D influences) here.

## Results

Descriptive statistics for each dimension of the ANPS for the total sample as well as separately for each group are provided in Table 2. Mean and variance differences among twin and sibling groups were inspected given that they can affect overall model fit [33]. For each ANPS factor the normal distribution could be assumed according to visual inspection, skewness and kurtosis statistics and the results of the Kolmogorov-Smirnov goodness-of-fit test (p-values between .13 and .95). Correlations between age and ANPS scales ranged between -.02 (for Spirituality) and .24 (*p* < .01; for PLAY). For FEAR (*t* (793) = 6.20; *p* < .01), CARE (*t* (793) = 9.27; *p* < .01), ANGER (*t* (793) = 3.41; *p* < .01), SADNESS (*t* (793) = 9.67; *p* < .01) and Spirituality (*t* (793) = 3.16; *p* < .01), females scored slightly to modestly higher than males. (For these ANPS scales, we also inspected twin and sibling resemblances for male and female pairs separately to see if the relative importance of genetic and environmental influences differs for male and female. For all scales, patterns of resemblances were comparable to those derived from to total twin and sibling groups indicating no meaningful gender differences with respect to the relative contribution of genetic and environmental influences. Therefore heritability was estimates based on the total sample.) After correction for age and sex, there were no statistically significant differences between group means and variances as determined by one-way ANOVAs and Levene’s tests for the residual scores of all ANPS dimensions. As can be seen in Table 3, correlations ranged between .00 and .66 for twin 1 as well as .01 and .62 for twin 2 indicating a large overlap between specific ANPS scales. Table 4 shows twin and non-twin sibling ICCs as well as p-value differences for ICCs between DZ twins and non-twin siblings. As can be seen, MZ twin correlations exceeded those of the DZ twin and non-twin sibling pairs in all cases. For FEAR, ANGER, PLAY and SADNESS, MZ twin correlations were over double the DZ correlations suggesting genetic dominance influences to be especially important. Regarding SEEK CARE and Spirituality, twin correlations rather pointed to shared environmental influences. Apart from the pattern of twin similarities, relatively high resemblances within non-twin siblings rather indicated shared environmental influences for all ANPS dimension except SEEK and SADNESS. Moreover, sibling resemblances were significantly different from the corresponding DZ twin resemblance for CARE. As described above, both genetic dominance and shared environment cannot be estimated simultaneously. Because of these somewhat ambiguous patterns of similarities, we compared models including shared environment or genetic dominance (based on AIC) for all ANPS dimensions. Model fit statistics for the full and best-fitting models as well as parameter estimates are shown in Table 5. Model fitting results showed good model fits for all univariate models compared to the saturated model.

**Table 4:** Twin and sibling resemblance (ICCs) for the Affective Neuroscience Personality Scale.

Measure	Resemblance			p-value difference btwn DZ and siblings^a^
	MZ	DZ	Siblings	
SEEK	.32**; (.17 - .46)	.22*; (.01 - .41)	.11; (-.07 - .28)	.22
FEAR	.50**; (.36 - .61)	.08; (-.13 - .29)	.30**; (.13 - .46)	.06
CARE	.63**; (.52 - .71)	.45**; (.27 - .61)	.19*; (.02 - .36)	.02*
ANGER	.43**; (.29 - .55)	.11; (-.11 - .31)	.30**; (.13 - .45)	.08
PLAY	.66**, (.56 - .74)	.05, (-.17 - .25)	.21**; (.04 - .37)	.13
SADNESS	.39**; (.25 - .52)	.17; (-.05 - .37)	.13; (-.05 - .30)	.39
Spirituality	.54**; (.41 - .64)	.38**; (.18 - .55)	.27**; (.10 - .43)	.20

*Note*: MZ = Monozygotic twins; DZ = Dizygotic twins; ^a^ Correlations between DZ twins and siblings were tested for significant differences, two-tailed testing

* p < .05

** p < .01. The numbers in brackets refer to the confidence intervals.

**Table 5:** Model-Fitting results and parameter estimations based on the extended classical twin design.

	Model	*χ*^*2*^	*df*	*p*	AIC	A	C/D	E
SEEK								
	ACE	8.90	11	.63	2704.29	.32	.00	.68*
	**ADE**	**8.86**	**11**	**.64**	**2704.24**	**.26**	**.07**	.67*
FEAR														
	ACE	6.86	11	.81	3160.10	.50*	.00	.50*
	**ADE**	**6.16**	**11**	**.86**	**3159.40**	**.30**	**.22**	.48*
CARE														
	ACE	8.92	11	.63	2791.14	.61*	.00	.39*
	**ADE**	**8.90**	**11**	**.63**	**2791.13**	.58*	**.03**	.39*
ANGER														
	**ACE**	**8.77**	**11**	**.64**	**3084.68**	.31*	**.09**	.60*
	ADE	9.16	11	.61	3085.07	.41*	.00	.59*
PLAY														
	ACE	27.31	11	.01	2858.71	.65*	.00	.35*
	**ADE**	**15.89**	**11**	**.15**	**2847.28**	**.01**	.68*	.31*
SADNESS														
	ACE	5.25	11	.92	2757.72	.38*	.00	.62*
	**ADE**	**4.60**	**11**	**.95**	**2757.07**	**.16**	**.24**	.60*
Spirituality														
	**ACE**	**7.05**	**11**	**.80**	**2982.94**	.52*	**.05**	.43*
	ADE	7.17	11	.78	2983.06	.57*	.00	.43*

*Note*: All parameter estimates are presented squared and fully standardized. A = additive genetic influences; D = non-additive genetic influences; C = shared environmental influence; E = non-shared environmental influence; AIC = Akaike’s information criterion

* *p*< .05; the preferred model is boldfaced.

First of all, models with different environmental estimates for twin and non-twin sibling pairs (i.e. assuming specific twin influences) did not fit the data significantly better than either of the models equating these influences. The final models for all ANPS scales favored equal environmental estimates across all groups. For ANGER and Spirituality an ACE model including additive genetic, shared and non-shared environmental influences yielded the best model fit while for the remaining dimensions an ADE model including additive and non-additive genetic as well as non-shared environmental influences fitted the data best. Fully standardized, heritability estimates (including additive and non-additive genetic influences) ranged from 33% for SEEK up to 69% for PLAY. Regarding FEAR, PLAY and SADNESS these genetic influences were in large part of a non-additive nature while SEEK and CARE showed only small proportions of non-additivity (between 3% for CARE and 68% for PLAY). With respect to the ACE models for ANGER and Spirituality, shared environmental influences were not significant and explained only 9%, respectively 5% of the variance. In comparison, non-shared environmental influences ranged between 31% (PLAY) and 67% (SEEK). Although internal consistencies for the ANPS scales were all no less than acceptable, any random measurement error affects estimates of genetic and environmental influences that typically lead to an underestimation of heritability [22]. Therefore, we further corrected heritability estimates (including additive and non-additive influences) for the corresponding reliabilities of the scales to get a more appropriate basis to compare them. (Heritability estimates from the model were standardized based on a variance of 1. To get estimates for the *true* variance corrected for measurement error, we used the following formula: h^2^_corr_ = h^2^/α) After correction, lowest heritability estimates were found for ANGER (37%) and SEEK (48%), followed by SADNESS (56%), FEAR (60%), and Spirituality (64%). Highest estimates appeared for CARE and PLAY (82% and 88%).

**Fig 1:**
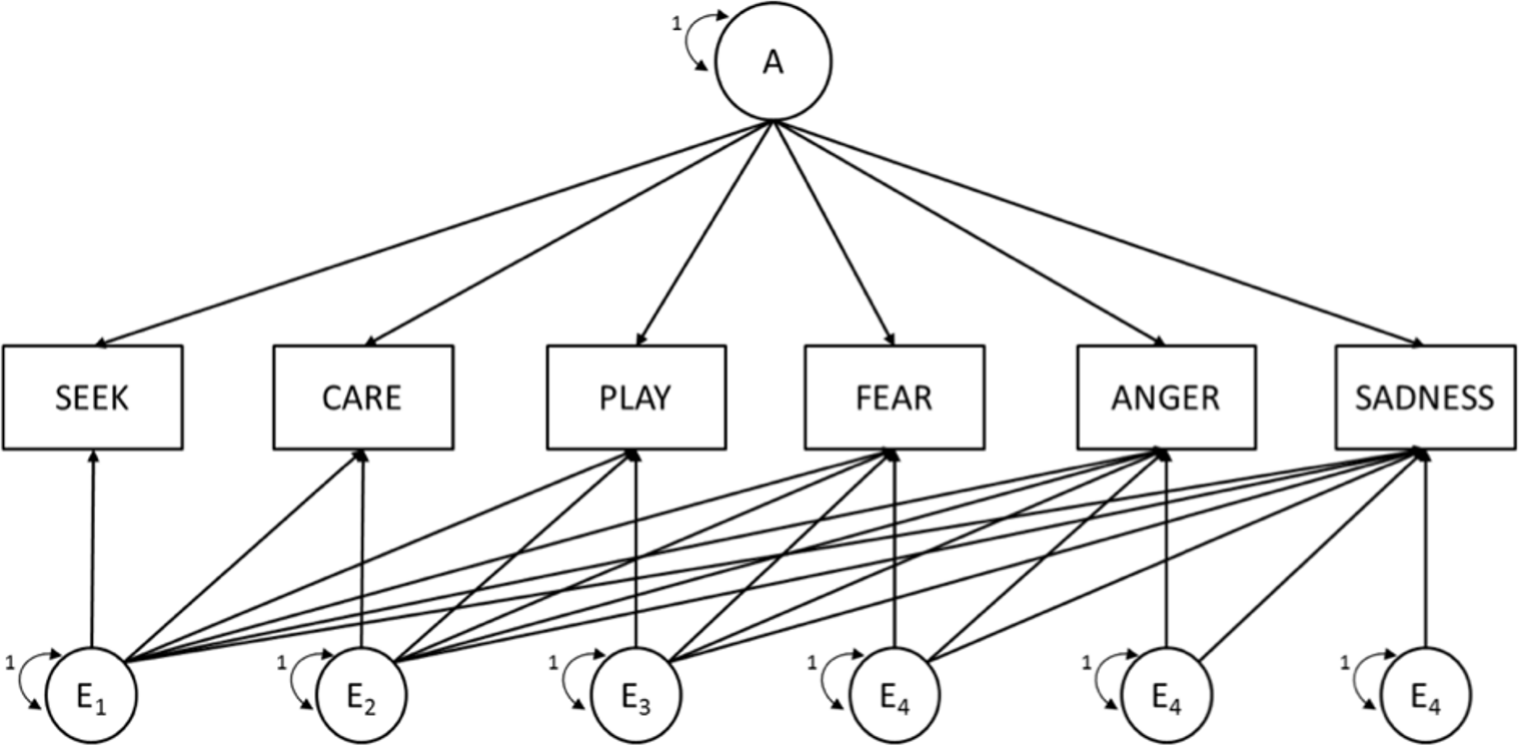
Multivariate independent pathway model of the relations between the six ANPS scales. The best fitting model, with an independent pathway specification for additive genetic influences (A), and a cholesky decomposition for non-additive genetic influences (D) and non-shared environmental influences (E). For a better illustration, the model only shows A and E influences. D influences are not shown in the Figure, but were modeled the same way as E influences using a cholesky decomposition. For simplicity, the model is shown only for one member of a pair.

The model fitting results for the multivariate genetic models are presented in Table 6. All multivariate genetic models were tested compared to the multivariate saturated model and showed no significant differences in overall model fit statistics. The full cholesky decomposition model including specific and common additive and non-additive genetic as well as non-shared environmental influences for each of the six ANPS scales and among them provided a good fit to the data. Compared to this ‘baseline’ model, common pathway models (Model 13 and 14) with phenotypic latent factors (one or two factors) did not describe the data well. Within the independent pathway models (Model 4–12), the best fitting model (Model 9; see Fig 1 for an illustration) included an independent pathway specification for additive genetic influences and a cholesky decomposition for non-additive genetic influences as well as for non-shared environmental influences.

**Table 6:** Fit statistics for multivariate genetic model fitting.

Model	-2LL	*df*	Compared to	*χ*^*2*^-diff	*df*	*p*	AIC
Saturated model	11049.74	4092					2865.74
ADE Cholesky decomposition	11186.99	4203	1	137.26	111	.06	2781.00
AE Cholesky decomposition	11211.01	4224	2	24.02	21	.29	2763.01
Independent pathway (common + specific ADE)	11294.14	4230	2	107.15	27	.00	2834.14
Independent pathway (common + specific AE)	11357.88	4242	2	170.89	39	.00	2873.88
Independent pathway (common AD + specific ADE)	11388.29	4236	2	201.30	33	.00	2916.29
Independent pathway (common A + specific ADE)	11558.27	4242	2	371.28	39	.00	3074.27
Independent pathway (common AD + specific AD + Cholesky E)	11224.14	4221	2	37.15	18	.01	2782.14
Independent pathway (common AD + Cholesky E)	11301.28	4233	2	114.28	30	.00	2835.27
**Independent pathway (common A + Cholesky DE)**	**11195.17**	**4218**	**2**	**8.17**	**15**	**.92**	**2759.17**
Independent pathway (2 common A + Cholesky DE)	11199.87	4218	2	12.87	15	.61	2763.87
Independent pathway (2 common AD + Cholesky E)	11292.00	4233	2	105.00	30	.00	2826.00
Common pathway ADE	11469.59	4240	2	282.60	37	.00	2989.59
Common pathway 2 latent factors ADE	11331.90	4235	2	144.90	32	.00	2861.90

*Note*. A = additive genetic influences; D = non-additive genetic influences; E = non-shared environmental influence; -2LL = -2 times Log-likelihood of data; *df =* degrees of freedom; AIC = Akaike’s information criterion; the preferred model is boldfaced.

Table 7 provides standardized coefficients of additive genetic, non-additive genetic and non-shared environmental influences on the variance of each scale as well as the covariation among the scales based on the best fitting model. The results showed that the additive genetic variance (between 1% and 19%) in each ANPS scale was common to all six scales and that there was no specific additive genetic variance for a specific ANPS scale. With respect to non-additive genetic influences, genetic correlations between the scales were small to moderate and ranged between -.55 and .52. This means that only a part of the non-additive genetic variation was common to the specific scales. The same pattern can be seen for the non-shared environmental influences. Environmental correlations ranged between -.16 and .51.

**Table 7:** Standardized estimates for additive genetic, non-additive genetic, and non-shared environmental influences on the six ANPS scales as well as genetic and environmental correlations based on the best fitting model (please see Fig 1 for additional information).

	A	D						E					
		SEEK	CARE	PLAY	FEAR	ANGER	SADNESS	SEEK	CARE	PLAY	FEAR	ANGER	SADNESS
SEEK	**.17**	**.17**						**.66**					
CARE	**.19**	-.45	**.43**					.24	**.38**				
PLAY	**.01**	.41	.27	**.69**				.41	.35	**.30**			
FEAR	**.15**	-.32	.52	-.55	**.39**			.02	.10	-.16	**.46**		
ANGER	**.05**	-.10	.24	.00	.31	**.37**		.06	-.06	-.10	.23	**.58**	
SADNESS	**.01**	-.32	.44	-.48	.90	.40	**.42**	-.02	.08	-.09	.51	.21	**.57**

*Note*. A = additive genetic influences; D = non-additive genetic influences; E = non-shared environmental influence; Standardized estimates for A, D and E influences are boldfaced; Correlations for non-additive genetic and non-shared environmental influences between the six scales are pictured below the diagonal; As modeled by a single independent factor, additive genetic correlation is 1 between all ANPS scales.

## Discussion

The present study aimed to investigate the influence of genetics and the environment on individual differences in ANPS-estimated primary emotional systems by means of a twin study. Our results show that every scale of the ANPS is influenced by genetics, but to varying degrees. The lowest heritability estimates are observed for the SEEK, ANGER and SADNESS system ranging between 31 and 40% (corrected 42 and 58%). Highest heritability estimates are observed for FEAR, CARE and PLAY going beyond .50. The genetic influence on individual differences in the PLAY system is especially pronounced (about .67; corrected .86). Thereby the results were comparable to findings of other twin studies using different personality inventories such as the Five Factor Model (see [17] for a review). Previous studies on the Big Five personality traits reported substantial genetic influences to a comparable degree. Moreover, for most of the Big Five personality traits, especially Neuroticism, Extraversion, Openness as well as Conscientiousness, there is substantial evidence for non-additive genetic influences [4,34]. Therefore, one explanation for the relation between PLAY and Extraversion and the connection of FEAR, ANGER and SADNESS with Neuroticism [9] could be that there is a genetic link including non-additivity between these constructs.

Moreover, a comparison of a variety of different multivariate genetic models provided first insights into genetic and environmental causes of phenotypic relations among the ANPS scales. The best fitting model showed an independent pathway specification for additive genetic influences and a cholesky composition for non-additive genetic as well as non-shared environmental influences. The finding of a single additive genetic component indicates that different primary emotional systems are not distinct at the level of additive genetic influences because all six scales loaded on one genetic factor. One explanation for this common genetic factor could be a similar set of genes. However, for non-additive genetic influences as well as non-shared environmental influences, correlations were small to moderate suggesting independent influences on specific emotional systems. So, although it can be assumed that genetic influences—mainly represented by a common genetic factor for all scales—are important, influences of non-shared environmental factors unique to each ANPS scale explain the remaining part of the variance. As the primary emotional systems could be seen as the basis of the Five Factor Model (e.g. PLAY underlying Extraversion or SEEK underlying Openness to Experience), similar non-environmental factors could play a role as observed in twin studies on the Five Factor Model. Such non-environmental variables being responsible for differences of the investigated persons of the same family could be “family composition, parental treatment, sibling interactions and extra-familial influences such as peers in addition to non-systematic factors. “([35]; p. 584)

Following from these findings the administration of the scales is of special value for molecular genetic studies (e. g. [36,37,38] These studies show that (an interaction of) dopaminergic genetic markers, but also an interaction of serotonergic and oxytocinergic markers are associated with individual differences in the primary emotional systems as measured with the ANPS), because a) an influence of genetics on individual differences of all primary emotional systems is demonstrated and b) the ANPS along with Panksepp’s cross species Affective Neuroscience approach to understanding primal emotions [1] now represents a genetically substantiated guide to test different brain transmitter systems and neuroanatomical structures (e. g. with MRI, [39]) in the context of each of the distinct primary emotional systems.

There are also limitations. Clearly, the questionnaire represents a cognitive approach to one’s own emotional experience; therefore it does not grasp emotional tendencies in a neuroscientifically direct way, e. g. as by directly observing human emotional behavioral and concurrent brain activities. This is put by Davis & Panksepp ([10], page 1952) as follows: “Although ANPS items attempt to address primary affects directly, since all self-report assessments must include cognitive reflection, we interpret the ANPS scales as tertiary (thought-mediated) approximations of the influence of the various primary emotional systems in people’s lives.”A second limitation concerns the sample size of the present twin study, which is relatively small. Previous studies have shown that some influences (e.g. shared environment) were often found to be non-significant due to small sample sizes and in consequence less power to detect them. In consideration of this issue, we decided to present the full model and not to exclude non-significant influences. However, compared to the classical twin approach, our sample was not limited just to twin data, which strengthens the assumption that the results are representative of the population. Third, there are some limitations that are inherent to most behavior genetic studies concerning different assumptions of the classical twin design (for an overview, see [21]). For example, the effect of gene-environment correlation and interaction could also be relevant in explaining individual differences in primary emotional systems, and hence should be considered in future research which requires information about specific environmental characteristics or specific genes.

In sum, the present study demonstrates that the ANPS is a new substantive empirical tool for biological oriented personality psychologists, which can advance the understanding of other major dimensions of human life. We anticipate the relevance of such understanding to eventually contribute to the study of imbalances of various primary emotional systems not only in various human addictions, but also a wide range of psychopathologies [40,41] especially various affective disorders (e.g. [2,3,8]).
