# Correction: The Role of Nature and Nurture for Individual Differences in Primary Emotional Systems: Evidence from a Twin Study

**DOI:** 10.1371/journal.pone.0157200

**Published:** 2016-06-03

**Authors:** Christian Montag, Elisabeth Hahn, Martin Reuter, Frank M. Spinath, Ken Davis, Jaak Panksepp

There is an error in affiliation 2 for authors Elisabeth Hahn and Frank M. Spinath. Affiliation 2 should be: Department of Psychology, Saarland University, Saarbrücken, Germany.

There are multiple errors in the text. There is an error in the third sentence of the Abstract. The correct sentence is: Lowest heritability estimates are presented for the ANGER system (31%) and highest for the PLAY system (69%).

There is an error in the fourth sentence of the second paragraph of the Results section. The correct sentence is: Fully standardized, heritability estimates (including additive and non-additive genetic influences) ranged from 31% for ANGER up to 69% for PLAY.

There is an error in the fifth sentence of the second paragraph of the Results section. The correct sentence is: Regarding FEAR, PLAY and SADNESS these genetic influences were in large part of a non-additive nature (between 22% and 68%) while SEEK and CARE showed only small proportions of non-additivity (between 3% and 7%).

There is an error in the seventh sentence of the second paragraph of the Results section. The correct sentence is: In comparison, non-shared environmental influences ranged between 31% (PLAY) and 69% (ANGER).

There is an error in the third sentence of the first paragraph of the Discussion section. The correct sentence is: The lowest heritability estimates are observed for the SEEK, ANGER and SADNESS system ranging between 31 and 40% (corrected between 37% and 58%).

There is an error in the fifth sentence of the first paragraph of the Discussion section. The correct sentence is: The genetic influence on individual differences in the PLAY system is especially pronounced (about .69; corrected .88).
